# Tailor-Made Surgery Based on Functional Networks for Intractable Epilepsy

**DOI:** 10.3389/fneur.2020.00073

**Published:** 2020-02-13

**Authors:** Kyousuke Kamada, Christoph Kapeller, Fumiya Takeuchi, Johannes Gruenwald, Christoph Guger

**Affiliations:** ^1^Department of Neurosurgery, Megumino Hospital, Eniwa, Japan; ^2^ATR Advanced Telecommunications Research Institute International, Kyoto, Japan; ^3^g.tec Guger Technologies OG/g.tec Medical Engineering GmbH, Schiedlberg, Austria; ^4^Department of Research Promotion Center, Asahikawa Medical University, Asahikawa, Japan

**Keywords:** drug resistant epilepsy, evoked potentials, electrocorticography, prognosis, surgery

## Abstract

Normal and pathological networks related to seizure propagation have got attention to elucide complex seizure semiology and contribute to diagnosis and surgical monitoring in epilepsy treatment. Since focal and generalized epileptogenic syndromes abnormalities might involve multiple foci and large-scale networks, we applied electrophysiolpgy (cortco-cortico evoked potential; CCEP), and tractography to make detailed diagnosis for complex syndrome. All 14 epilepsy patients with no or little abnormality on images investigations underwent subdural grid implantation for epilepsy diagnosis. To perform quick network analysis, we recorded and analyzed high gamma activity (HGA) of epileptogenic activity and CCEPs to identify pathological activity distribution and network connectivity. [Results] Pathological CCEPs showed two negative deflections consisting of early (>40 ms) and late (>150 ms) components in electrically stable circumstance at bed side and early CCEPs appeared in 57% of the patients. On the basis of the CCEP findings, tractography detected anatomical connections. Early components of pathological CCEPs diminished after complete disconnection of tractoography-based fibers between the foci in seven of eight cases. One case with residual pathological CCEPs showed poorer outcome. Thirteen (92.8%) patients with or without CCEPs who underwent network surgery had favorable prognosis except for a case with wide traumatic epilepsy. Intraoperative CCEP measurements and HGA mapping enabled visualization of pathological networks and clinical impotence as a biomarker to improve functional prognosis. HGA/CCEP recording should shed light on pathological and complex propagation for epilepsy surgery.

## Introduction

Intractable epilepsy frequently involves multiple pathological foci and networks (1–3). Epileptic activity involving pathological networks may be developed by frequent ictal states and promote pathological neural plasticity that further strengthens epileptic networks and connectivity (1–3). Analyzing complex epileptic foci and networks could reveal pathological mechanisms underpinning seizure initiation and propagation (2). In order to identify the seizure propagation. As we mentioned above, we demonstrate that the epileptic brain consists of foci and subcortical functional networks (3, 4).

A representative keyword in epilepsy diagnosis is “high frequency” which includes fast ECoG components higher than 100 Hz and has already become a pathological biomarker for the identification of epileptogenic foci (5, 6). Recently, high gamma activity (HGA) between 70 and 160 Hz on ECoG has received great attention for achieving functional mapping for the eloquent cortices using different semantic tasks (7–9). Thus, it is expected that HGA analysis would enable the visualization of relationships between eloquent and pathological cortical areas. Besides the development of HGA-cortical mapping, it is still difficult to discriminate primary from secondary foci or seizure initiation and propagation by HGA-correlation analysis. Several investigators have reported phase synchrony on multiple cortices with different frequency bands to identify subcortical connections (10, 11), and it was technically difficult to validate the findings in the present clinical practice. Another image technique for network analysis is diffusion-weighted imaging (DWI) based tractography and resting state functional MRI (rs-fMRI) have shown potential for revealing the implicated pathological network (1, 12, 13). Presently, tractography can visualize a limited number of subcortical fibers because of the simple fiber-tracking theory based on anisotropic tensor values (14) and complex anatomical structures for neurosurgery. Conversely, networks involving pathological activities have not been well-investigated. For epilepsy diagnosis, DTI-based tractography identifies a few major bundles related to language and motor functions (13, 15, 16), but it is insufficient to make clear discrimination between normal and pathological fibers. It is thus becoming clear that functional networks involving epileptogenic foci facilitate seizure initiation and propagation. Electrocortical stimulation with alternative currents to the eloquent or epileptogenic cortical regions induced cortico-cortical evoked potentials (CCEPs) in the functionally-connected areas. CCEP recordings have received attention for the identification and monitoring of subcortical functions compared to other neuroimaging modalities. Recent studies have shown that bedside-CCEP mapping is reliable for the monitoring of subcortical functions and the identification of new networks, validated by ECS mapping (17, 18). Furthermore, few studies have applied CCEP to identify pathological functional networks in epilepsy (3, 4, 18). Previous reports have described that CCEPs indicate normal connections mediating motor and language-related functions (17) and could be recorded intraoperatively as a biomarker of functional connectivity of eloquent brain functions. CCEPs at the bedside consisted of two major deflections, N1 and N2, peaking at ~30 and 100 ms, respectively. Compared to the later N2 component, N1 might reflect direct connections from the stimulus points and be relatively stable according to previous studies (17). Furthermore, a recent report described profiles of CCEPs evoked by stimulus to the foci to show propagation of epileptic discharges (pathological CCEP) within the motor system (19, 20). They speculated that the spread of epileptic activity within the networks is a predictive negative factor for functional outcome (19). Therefore, CCEP monitoring by normal and epileptogenic foci stimuli might be a key tool to identify normal and aberrant connections that could contribute to predicting seizure outcome and elucidating pathophysiology in epilepsy. In this study, we performed intraoperative CCEP monitoring by eloquent areas and the foci to identify normal and pathological networks. Furthermore, selective fiber disconnection based on intraoperative CCEP profiles and tractography were used to develop a tailor-made surgical strategy for intractable epilepsy. The combination of novel techniques enable us to understand the epilepsy pathology and improve functional prognosis of patients.

## Materials and Methods

### Patients

The research ethics board (internal reviewing board: IRB) approved the study (No. 183). Written informed consent was obtained from all subjects and their families. Inclusion criteria were frontal or temporal epilepsy with no or little abnormal signal intensity on MRI and exclusion was severe brain damage by stroke or meningitis.

**Figure 1:**
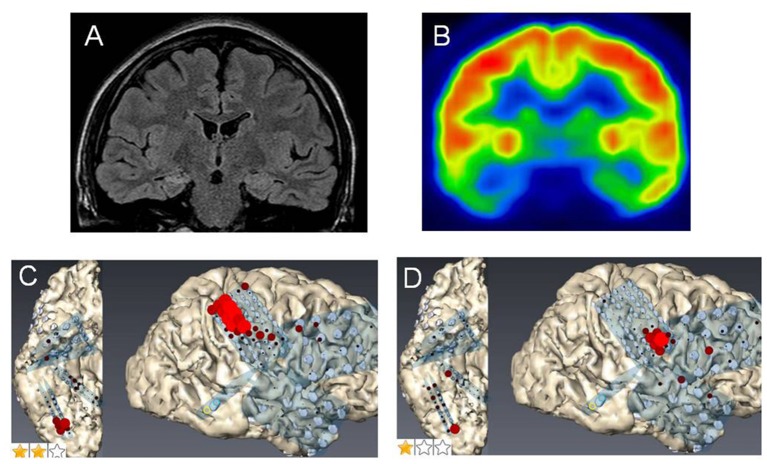
Case 2: **(A)** Fluid attenuation inversion recovery imaging showing mild right hippocampal sclerosis. **(B)** Fluorodeoxyglucose-positron emission tomography revealing a hypometabolic lesion in the right mesial and lateral temporal regions. High gamma activity (HGA) mapping indicating hand- **(C)** and tongue- **(D)** primary motor areas (PMAs).

Fourteen patients with intractable epilepsy who had undergone subdural grid implantation for diagnostic purposes between April 2012 and September 2018 (Table 1) participated in this study. Eight patients lacked gross structural brain abnormalities. Eight of 14 patients had no abnormality on anatomical MRI. Cases 1, 2, and 14 showed wide posttraumatic scarring over the left posterior-temporal region and obvious cortical dysplasia, respectively, which indicated focal cortical dysplasia (FCD) II on anatomical MRI. Three cases (3, 4, and 5) had hippocampal sclerosis in the right mesial temporal lobe (Figure 1). Twenty-four-hour video–EEG monitoring for seizure diagnosis detected pathological spikes and activities associated with the special seizure semiology. No patient had infection or hemorrhagic complications.

**Table 1:** Basic clinicodemographic characteristics of individual patients.

**Case**	**Age**	**Gender**	**Foci**	**Pathology**	**MRI findings**	**Symptoms**
1	30	Female	Rt T	Trauma	Lt posterior temporal	CPS/GCS
2	22	Male	Lt F	FCD II	Rt motor cortex	GCS
3	27	Male	Rt T	HS	+	CPS/GCS
4	24	Male	Rt T	HS	+	CPS/GCS
5	28	Male	Rt T	HS	+	CPS/GCS
6	33	Female	Rt T	FCDI	none	CPS/GCS
7	33	Female	Rt T	none	none	CPS
8	32	Female	Lt T	FCD I	none	CPS
9	29	Female	Lt F/T	FCD I	+	CPS
10	31	Male	Lt T	none	none	CPS
11	28	Male	Bil F	none	+	CPS/GCS
12	31	Male	Rt T	none	none	CPS/GCS
13	16	Female	Rt T	none	+	CPS/GCS
14	50	Female	Bil F	FCD II	+/mild atrophy	CPS/Drop attack

*CPS, complex partial seizure; FCD l, focal cortical dysplasia type l; FCD II: focal cortical dysplasia type ll; HS, hippocampal sclerosis; GCS, generalized convulsive seizure; Lt F/T, left fronto-temporal; Bil F, bilateral frontal; Lt T, left temporal; Rt T, right temporal; [+] of MRI abnormality indicates hyperintensity or atrophy in the medial temporal regions*.

### Preoperative Imaging Studies

All 14 patients underwent preoperative three-dimensional structural imaging and DTI-MRI using a 3.0-Tesla whole-body MRI scanner (Discovery 750 W; General Electric, Milwaukee, WI, USA) to investigate structural associations based on electrophysiological analysis. We obtained 55 images with 3-mm thickness by echo-planar imaging with a *b*-value of 1,000 s/mm^2^. For each subject, a high-resolution anatomical dataset was also acquired by a three-dimensional spoiled gradient recalled echo sequence (3D-MRI). Probabilistic tractography was performed using the FMRIB diffusion toolbox (FDT v2.0, http://fsl.fmrib.ox.ac.uk/fsl/fslwiki/FDT). BEDPOSTX was used to model 5,000 iterations within each voxel at a curvature threshold of 0.2, step length of 0.5, and maximum step number of 2,000. Target masks were used (the cerebral peduncle, motor and language areas, and epileptogenic foci) and the distribution of fiber orientations was calculated between pairs of masks (functionally connected areas). The seeds for fiber tracking were placed on voxels overlapping subdural grid channels that induced normal or pathological CCEPs.

### Video–ECoG Monitoring

We implanted intracranial electrodes with a 3-mm electrode diameter and 10-mm inter-electrode distance (Unique Medical, Tokyo, Japan) on wide brain areas including the suspected epileptogenic foci and a 4-channel strip for the reference and ground channels. More than three seizures were captured from each patient to identify epileptogenic dynamics. Seven patients showed a single epileptogenic focus. Five, however, had multiple foci within one hemisphere and two exhibited multiple foci in the bilateral hemispheres.

### HGA Mapping

After video-ECoG monitoring to identify the epileptogenic foci, all patients underwent HGA mapping and CCEP recording using all ECoG channels. The intracranial recordings were performed using a 256-channel g.HIamp bio-signal amplifier (Guger Technologies GmbH, Graz, Austria).

### Functional and Epilepsy Mapping by HGA Analysis

#### CCEP Recording for Functional Connectivity

We recorded CCEPs with electrical stimulation to different places including the eloquent areas and epileptogenic foci to identify functionally-connected brain regions. The stimulus condition was a constant current square wave pulse of 0.3 ms duration delivered at 1 Hz. In each session, we performed two CCEP measurements with alternative bipolar stimulation to confirm reproducibility. The baseline and post-stimulus recording periods were −100 and 800 ms related to the trigger, respectively. The Welch-test, a derived *t*-test for populations with different variances, was used to check for significant differences between the averaged baseline and CCEP waveforms. We paid the most attention to assign and confirm consistent CCEP findings. CCEP waveforms generally demonstrate 2 negative peaks (N1 and N2) by alternative-polarity cortical stimuli to suspected foci or non-focal regions. Latencies of N1 and N2 are ~ 30 and 100 ms, respectively. Furthermore, we accepted only CCEPs, which appeared on different electrodes from stimulation electrodes to avoid electrical artifect.

### Intraoperative CCEP Monitoring

We treated the patients with a refined monitored anesthesia care protocol consisting of a combination of local anesthesia. Drug infusion doses during operation ranged between 0.8 and 2.35 mg/kg/h for propofol and 0.04 and 0.08 μg/kg/min for remifentanil. In addition, the bispectral index was constantly maintained between 70 and 80 to detect CCEPs under general anesthesia. We continuously monitored CCEPs evoked by stimulation to the main epileptogenic focus (pathological CCEPs) during surgical resection. CCEP monitoring was performed on regions to be targeted for surgical resection.

## Results

### Video-ECoG Monitoring

The monitoring detected more than two seizures in all patients. Case 1 with wide cortical injury by brain trauma showed multiple spikes on more than 10 channels. We, therefore, select a stimulus channel pair, which evoked the highest pathological CCEP amplitudes on the channels. Other patients with or without MRI abnormalities, whose semiology suggested the frontal or/and the temporal lobe. ECoG demonstrated the epilepsy onsets and the suspected channel pairs were selected for stimulation to obtain little or the highest CCEPs.

### CCEPs by Stimulation to Epileptogenic Foci (Pathological CCEP)

After stimulation to the epileptogenic foci, we observed the CCEP profiles on the channels of all subdural grids. Cases 1 with posttraumatic epilepsy showed small, but widespread CCEPs on the posterior temporal and a part of occipital regions. Case 2 with focal FCD in the central region and five of the 10 cases of temporal lobe epilepsy (TLE) with stimulation to epileptogenic foci revealed no CCEPs. In contrast, the other five cases of TLE demonstrated pathological CCEPs on the ipsilateral (left) frontal (*n* = 1), lateral aspect of the temporal (*n* = 3), and posterior temporal base (*n* = 1) by stimulation to the mesial hippocampal regions (Table 2). In our study, we found only 50% of epilepsy cases. It is, therefore, important to assign and confirm the reproducible pathological results carefully. We have to keep the fact in our mind that stress that some epilepsy cases would demonstrate clear pathological CCEPs Concerning the latencies of CCEPS, we do more detailed analyzed, correcting more number of patients. Two cases with bilateral FLE had seizure initiation from both frontal areas. Since we determined that both frontal lobes were involved in the seizure onset, we stimulated multiple frontal points to evoke CCEPs on the frontal areas contralateral to the stimuli. Both these cases demonstrated obvious high amplitudes of CCEPs with bidirectional connections. Six cases of TLE and two cases of FLE demonstrated pathological CCEPs in functionally-connected areas. Although case 9 had the main seizure focus on the lateral aspect of the left temporal lobe, the pathological CCEPs appeared on the left frontal areas by stimulation of the temporal focus.

**Table 2:** Stimulus- pathological CCEP latencies in eight cases.

**Pathological CCEP**	**Stimulus point**	**No. of electrodes**	**Mean latency (ms)**	**Location of CCEP**
Case 3	Right hip	5	28.2 ± 1.5	Right temporal cortex
Case 4	Right hip	6	24.4 ± 1.5	Right temporal cortex
Case 5	Right hip	4	18.1 ± 0.8	Right posterior temporal base
Case 9	Left frontal	4	22.2 ± 0.7	Left temporal lobe
Case 11	Bil frontal	8	31.2 ± 2.7	Bil frontal lobe
Case 13	Right temporal	4	20.2 ± 1.4	Right temporal lobe
Case 14	Bil frontal	12	35.0 ± 4.7	Bil frontal lobe

*Bil, Bilateral; hip, hippocampus; CCEP, cortico-cortico evoked potential*.

### Profiles of Pathological CCEPs by Stimuli to the Epileptogenic Fpoci

Eight of the 14 cases were positive for pathological CCEPs (57%). Profiles of pathological CCEPs generally consisted of two major deflections with the first and second deflections peaking ~25 and 100 ms after the stimulation.

In patients with epilepsy with pathological CCEPs by stimulation to epilepileptogenic foci, we initially resected subcortical fibers between the stimuli and functionally connected CCEP-positive lesions until we confirmed that the CCEPs diminished. Whereas in cases with no CCEPs, we simply resected the epileptogenic focus, e.g., the hippocampus, FCD, or damaged brain lesion. We would like to demonstrate representative cases below. In order to minimize electrode positions, we continuously observe electrode positions, surface structures and little noise on raw ECoG.

**Figure 2:**
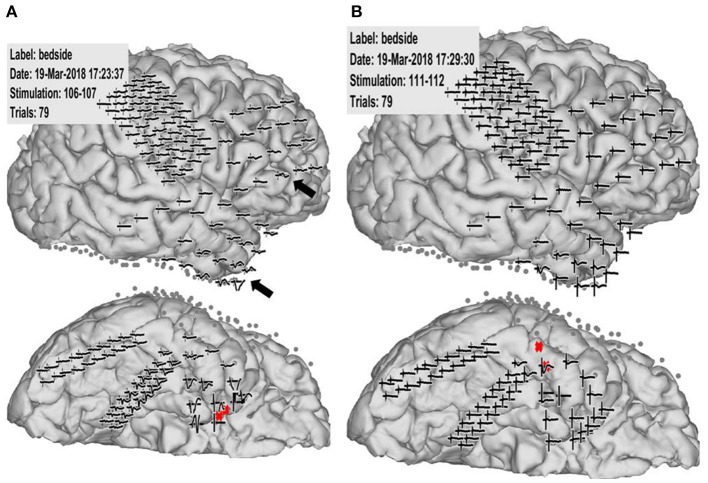
Case 2: Pathological cortico-cortico evoked potential (CCEP) results. **(A)** Right hippocampal stimulation (red circle) showing pathological CCEPs in the anterior and inferior frontal and anterior temporal lobes (black arrow). **(B)** Stimulation to the lateral part of the temporal base showing no response or electrical artifacts except during stimulus onset.

**Figure 3:**
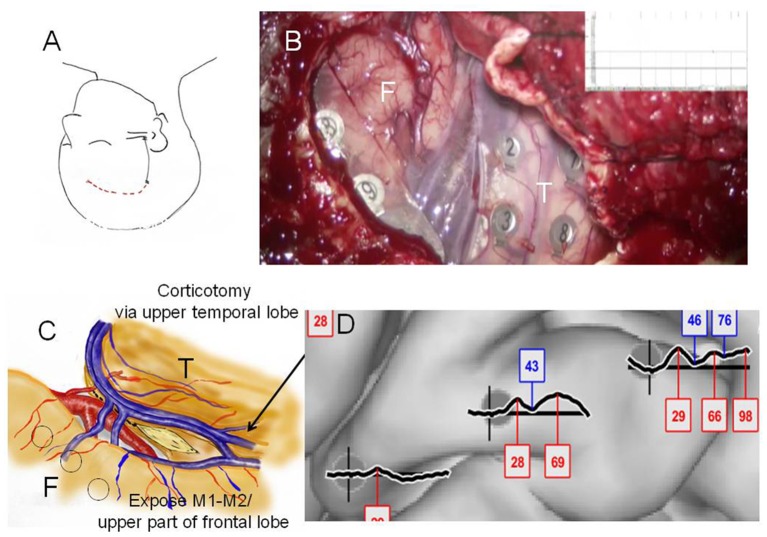
Case 2: **(A)** Initial skin incision for electrode implantation and second skin incision (epilepsy surgery) are indicated by the black and red lines, respectively. **(B)** Intraoperative photograph during the second surgery showing the frontal (F) and temporal (T) lobes, and Sylvian veins with implanted subdural electrodes for cortico-cortico evoked potential (CCEP) recording. (C) Operation sketch exposing the anterior and medial roofs of the temporal horn and shifting of the middle cerebral arteries to the frontal site (before disconnection). Black circles indicate CCEP-positive channels. **(D)** CCEP profiles on the right frontal lobe showing several deflections at approximately 30 and 70 ms.

**Figure 4:**
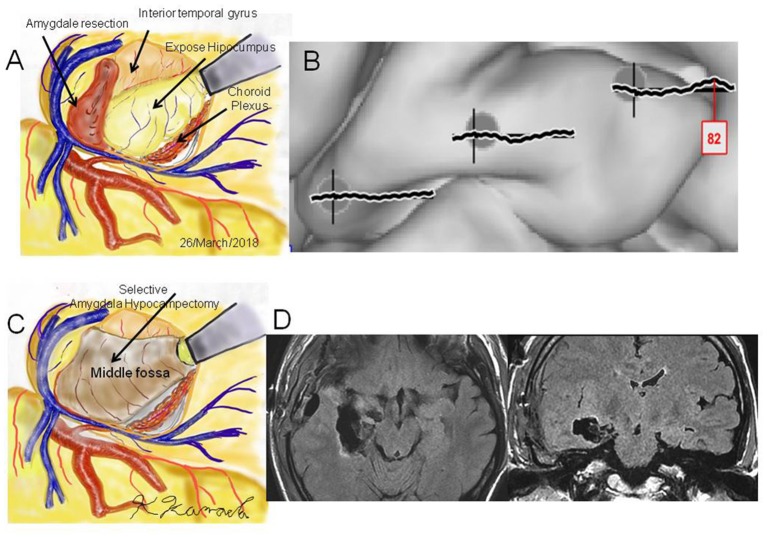
Case 2: **(A)** Operation sketch of disconnection of the Uncinate fascicles and removal of the anterior and medial roofs of the temporal horn. The hippocampus and choroid plexus were widely exposed. **(B)** Intraoperative cortico-cortico evoked potentials becoming extinct by hippocampal stimulation. **(C)** Operation sketch of complete hippocampectomy. **(D)** Postoperative magnetic resonance imaging showing selective hippocampectomy *via* the anterior ventral roof.

**Case 3** was a 23-year-old right-handed man who has seizures, which were preceded by autonomic and acoustic auras and frequently became generalized. Radiological examinations showed right hippocampal sclerosis on fluid attenuation inversion recovery imaging and hypometabolism in the anterior medial and lateral temporal regions on fluorodeoxyglucose-positron emission tomography (Figures 1A,B). Video–EEG monitoring showed frequent right-dominant fronto-temporal spike propagation. ECoG monitoring revealed two foci in the right frontal lobe and the right mesial temporal region, including the parahippocampal gyrus. HGA mapping indicated that there was red bubble accumulation in the hand and mouth primary motor areas (Figures 1C,D). We stimulated the brain surface using all electrode channel pairs to detect CCEPs. Stimulation of the hippocampus evoked pathological CCEPs in the anterior fronto-temporal regions (Figure 2). We used pairs of 8-channel T-shaped grids to continuously stimulate the left anterior parahippocampal gyrus (hippocampal stimulation). We dissected the Sylvian fissure and exposed the limen insula. The key procedure was to widely expose the upper and anterior roofs of the temporal horn after removal of the anterior part of the insular cortex. We finally exposed a 25-mm section of the left ventricle roof, indicating anatomical disconnection of the Uncinate fascicles (Figure 3). After disconnection and exposure of the hippocampus, pathological CCEPs disappeared (Figure 4). We thus performed selective hippocampectomy (Figures 4C,D). The patient was ultimately seizure-free with no neurological deficits during the past 3 years after the surgery.

**Figure 5:**
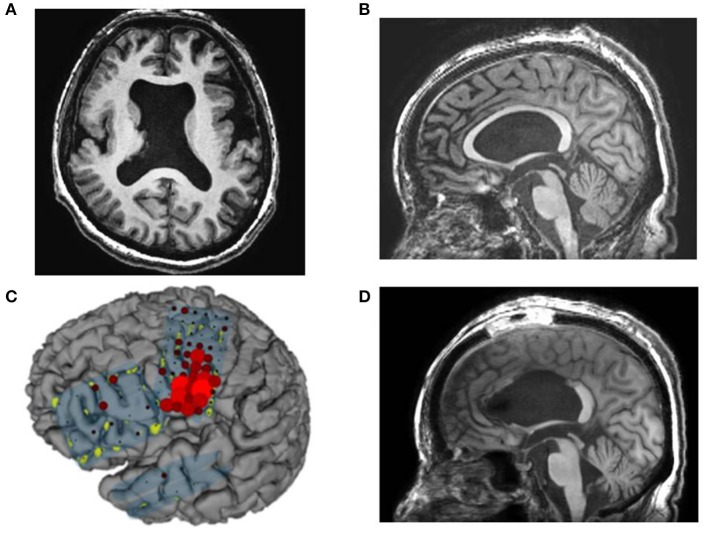
Case 14: **(A,B)** Preoperative magnetic resonance imaging showing cortical dysplasia and thin corpus callosum, **(C)** high gamma activity (HGA) mapping with an overt word reading task showing accumulation of HGA mainly on the mouth primary motor area (PMA). **(D)** Postoperative magnetic resonance imaging showing resection of the anterior and middle corpus callosum except for the splenium.

**Figure 6:**
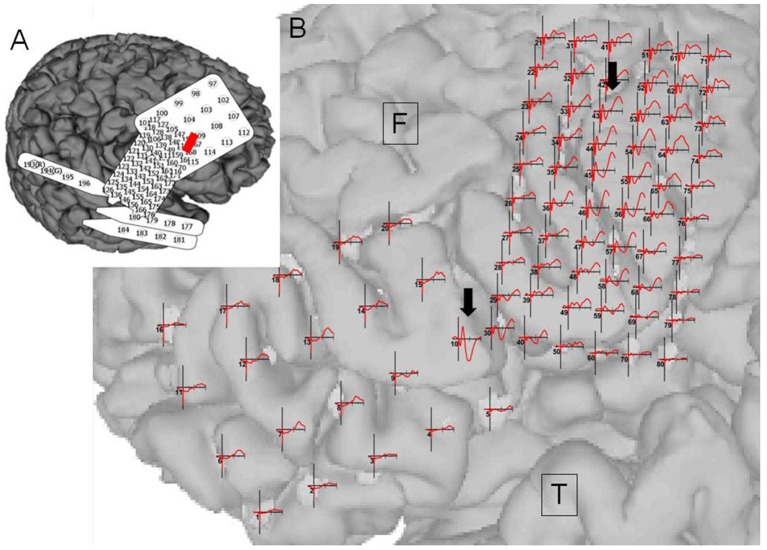
Case 14: **(A)** Electrode configuration of the right hemisphere showing the stimulus channel, which evoked the highest cortico-cortico evoked potential (CCEP) on the contralateral frontal lobe. **(B)** CCEPs by stimulation on the left primary motor area and the inferior frontal gyrus. Red circles and black arrows indicate the stimulus points and prominent CCEPs.

**Figure 7:**
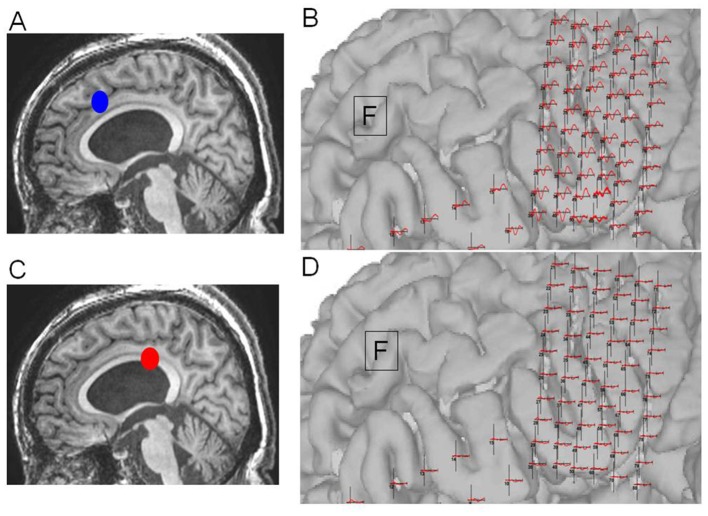
Case 14: **(A)** Initial operation step to expose the corpus callosum. **(B)** Pathological cortico-cortico evoked potential (CCEPs) still appearing before resection. **(C)** Corpus callosotomy approaching the splenium. **(D)** Pathological CCEPs diminishing after corpus callosotomy. Blue and red dots indicate the operation steps before and after resection, respectively.

**Case 14** had FCD type II in most cortical areas as shown on MRI and had daily drop attacks, CPS, and secondary generalization (Figure 5). Since she wished to undergo palliative treatment for her seizures, we first implanted subdural grids to reveal the semiology and electrophysiological findings. HGA mapping with an overt word reading task showed accumulation of high frequency oscillations mainly on the facial PMA (Figure 5C). Epileptic activities on ECoG hardly determines which side was dominantly involved. A channel pair on the right epileptic frontal focus caused “pathological CCEPs on the contralateral (left)” PMA and inferior frontal lobe (Figure 6). We, therefore, decided to perform corpus callosotomy. We continuously monitored the pathological CCEPs from the left PMA with right frontal stimulation to the foci. After we achieved four fifths of corpus callosotomy, the pathological CCEPs immediately diminished (Figures 5D, 7). Her postoperative course was uneventful. She was able to walk and went back to home and performed activities of daily living without support.

Engel class I outcome was achieved in 92.8% of patients (13/14 cases). Aura, the absence of secondary generalization, initial ictal patterns, and interictal EEG findings were correlated with favorable 1-year postsurgical outcome. Based on these, preoperative CCEP recording may reveal pathophysiological phenomena and suggests that a tailor-made surgical strategy should be employed for each patient.

## Discussion

Pathological CCEPs by electrical stimulation to foci appeared in 57% of patients with intractable epilepsy, while the other 43% has no pathological CCEPs. In the cases with pathological CCEPs, we further utilized intraoperative CCEP monitoring and observed the complete disappearance of pathological CCEPs after fiber disconnection in our previous report (2). In this report, we found amplitudes of pathological CCEPs by network disconnection. We would like to propose that pathological CCEPs could be a new biomarker for epilepsy surgery. Conversely, case 1 with posttraumatic epilepsy might have had insufficient coverage of subdural grids and pathological CCEPs remained. Intraoperative CCEP monitoring with sufficient area coverage of underlying pathology and would be a intraoperative biomarker of epilepsy surgery.

Tamura and our group described passive HGA/CCEP mapping for functional mapping during awake craniotomy (21). Electrical stimulation to the identified temporal receptive language area by linguistic-HGA mapping evoked CCEPs in the frontal language area. ECS confirmed that HGA/CCEP mapping regions were highly reliable for normal functional mapping (21, 22). Compared to previous intraoperative normal functional mapping, we further concentrated on identifying pathological networks in epilepsy by applying electrical stimuli to each focus at the bedside and in the operating room. We should remain cautious for the coverage areas of electrodes and electrical artifacts of single-pulse stimuli, which interfered with interpretation of the obtained results (23, 24). Electrical artifacts may most critically cause misunderstanding and confusion in CCEP results. The most careful point is that we accepted only responses from electrodes that differed from the electrode involving the stimulus channels. Intraoperative CCEPs with minimal artifacts to enable identification and confirmation of complete resection of pathological networks with strong confidence.

According to the interhemispheric or intrahemispheric connections, non-invasive procedures of transcranial magnetic stimulation and contralateral EEG showed that there might be bidirectional CCEPs between the primary motor cortices (25, 26). In these reports, the latencies between the bilateral PMA were 10 and 30 ms. Two papers described interhemispheric CCEPs by intracranial recording. Umeoka et al. found that there were several interhemispheric connections using CCEPs in five cases with bilateral basal temporal region involvement (27). In that report, CCEPs were recorded from a total of 24 electrodes after stimulation of the contralateral basal temporal regions. The latencies ranged from 48.2 to 102.3 ms (mean, 65.5 ms) for negative peaks and 70.2–122.0 ms (mean, 95.2 ms) for positive peaks. Terada et al. found obvious direct connections between both facial motor areas, but not between the sensory cortices. CCEPs by stimulation to the contralateral facial motor area peaked at ~30 and 50 ms (28). According to previous CCEP analyses, one can expect that thereare functional connectivities between both temporal and frontal lobes in epilepsy cases.

In this paper, we additionally found pathological CCEPs on the frontal and temporal lobes via intrahemispheric connectivity. Based on pathological CCEP profiles, we performed selective fiber disconnection in the Uncinate, inferior longitudinal fascicles, and corpus callosum. The disconnection of these fibers showed complete CCEP extinction based on tractography. We would like stress that this combination of CCEP recording and tractography could be a novel diagnostic strategy for epilepsy surgery to obtain favorable functional prognosis. The majority (13 among 14 cases with successful resection, 92.8%) showed favorable functional outcome. Thus, We would like to propose that pathological CCEPs could be a new biomarker for epilepsy diagnosis and intraoperative surgical decisions we would like to propose that combined CCEP monitoring, HGA mapping, and tractography may be advantageous for epilepsy diagnosis and warrants larger-scale application and evaluation.

## Data Availability Statement

All datasets generated for this study are included in the article/supplementary files.

## Ethics Statement

The studies involving human participants were reviewed and approved by The research ethics board (internal reviewing board: IRB) in Asahikawa Medical University (No. 183). The patients/participants provided their written informed consent to participate in this study.

## Author Contributions

KK: instructions of research flow, research designs, and operations. CK and FT: design of paradigms, and data processing. JG: design of paradigm and programming. CG: research organization and device development.

### Conflict of Interest

KK was employed by company ATR Advanced Telecommunications Research Institute International, Kyoto, Japan. CG is the CEO of g.tec medical engineering GmbH and JG and CK are employees of the company. The remaining author declares that the research was conducted in the absence of any commercial or financial relationships that could be construed as a potential conflict of interest.
